# Interferon lambda polymorphisms associate with body iron indices and hepatic expression of interferon-responsive long non-coding RNA in chronic hepatitis C

**DOI:** 10.1007/s10238-016-0423-4

**Published:** 2016-04-28

**Authors:** Anna Wróblewska, Agnieszka Bernat, Anna Woziwodzka, Joanna Markiewicz, Tomasz Romanowski, Krzysztof P. Bielawski, Tomasz Smiatacz, Katarzyna Sikorska

**Affiliations:** 1 Laboratory of Molecular Diagnostics, Intercollegiate Faculty of Biotechnology UG and MUG, Abrahama 58, 80-307 Gdańsk, Poland; 2 Department of Infectious Diseases, Pomeranian Center of Infectious Diseases, Smoluchowskiego 18, 80-214 Gdańsk, Poland; 3 Department of Infectious Diseases, Medical University of Gdansk, Smoluchowskiego 18, 80-214 Gdańsk, Poland; 4 Department of Tropical Medicine and Epidemiology, Medical University of Gdansk, Powstania Styczniowego 9b, 81-519 Gdynia, Poland

**Keywords:** Hepatitis C, chronic, Interferon lambda, Iron overload, Polymorphism, single nucleotide, RNA, long non-coding

## Abstract

Single nucleotide polymorphisms (SNPs) within DNA region containing interferon lambda 3 (*IFNL3*) and *IFNL4* genes are prognostic factors of treatment response in chronic hepatitis C (CHC). Iron overload, frequently diagnosed in CHC, is associated with unfavorable disease course and a risk of carcinogenesis. Its etiology and relationship with the immune response in CHC are not fully explained. Our aim was to determine whether *IFNL* polymorphisms in CHC patients associate with body iron indices, and whether they are linked with hepatic expression of genes involved in iron homeostasis and IFN signaling. For 192 CHC patients, four SNPs within *IFNL3*-*IFNL4* region (rs12979860, rs368234815, rs8099917, rs12980275) were genotyped. In 185 liver biopsies, histopathological analyses were performed. Expression of five mRNAs and three long non-coding RNAs (lncRNAs) was determined with qRT-PCR in 105 liver samples. Rs12979860 TT or rs8099917 GG genotypes as well as markers of serum and hepatocyte iron overload associated with higher activity of gamma-glutamyl transpeptidase and liver steatosis. The presence of two minor alleles in any of the tested SNPs predisposed to abnormally high serum iron concentration and correlated with higher hepatic expression of lncRNA NRIR. On the other hand, homozygosity in any major allele associated with higher viral load. Patients bearing rs12979860 CC genotype had lower hepatic expression of hepcidin (*HAMP*; *P* = 0.03). *HAMP* mRNA level positively correlated with serum iron indices and degree of hepatocyte iron deposits. *IFNL* polymorphisms influence regulatory pathways of cellular response to IFN and affect body iron balance in chronic hepatitis C virus infection.

**Electronic supplementary material:**

The online version of this article (doi:10.1007/s10238-016-0423-4) contains supplementary material, which is available to authorized users.

## Introduction

Chronic hepatitis C virus (HCV) infection affects more than 170 million people worldwide. Liver cirrhosis and hepatocellular carcinoma which develop as a consequence of chronic hepatitis C (CHC) are the leading causes of death in western countries. It is expected that HCV-related morbidity will increase during next 10–20 years [1].

In 2009, genome-wide associations studies identified three single nucleotide polymorphisms (SNPs) within DNA region containing interferon lambda 3 (*IFNL3*) and *IFNL4* genes, rs12979860, rs809917 and rs12980275, as prognostic factors of HCV-related liver disease, strongly associated with treatment-induced and spontaneous clearance of HCV [2–4]. Homozygosity in the major rs12979860 C allele in CHC patients correlates with a higher baseline viral load, lower expression of interferon-stimulated genes (ISGs) [5] and an increased rate of sustained virological response (SVR) [6]. Additionally *IFNL3*-*IFNL4* SNP variants are linked with metabolic abnormalities observed in CHC. The beneficial rs12979860 CC and rs8099917 TT genotypes are associated with a lower incidence of hepatic steatosis, higher serum LDL and total cholesterol levels in CHC but also with higher probability of hepatic inflammation and fibrosis [7–9]. Recently a genetic variant rs368234815 (ss469415590, ΔG/T) was found in strong linkage disequilibrium (LD) with rs12979860. Patients homozygous for T allele in rs368234815 show no expression of *INFL4* due to a disrupted open reading frame, in contrast to IFNL4-synthesizing carriers of the wild-type ΔG allele [10]. Hepatic expression of *IFNL4* is specifically induced upon HCV infection, and the presence of an active IFNL4 protein seems to be disadvantageous for disease course. It was suggested that rs368234815 is the causal SNP for the observed association between IFNL genotypes, HCV clearance and therapeutic outcome in CHC [11].

Dysregulation of iron homeostasis, which is diagnosed in up to 40 % cases of CHC and 50 % cases with both CHC and hepatocellular carcinoma, is associated with liver damage and correlates with a raised activity of aminotransferases, exacerbation of inflammation, progression of liver fibrosis, increased risk of hepatocarcinogenesis, and a decrease in the effectiveness of antiviral therapy [12–16]. However, the relationship between elevated iron indices, HCV life cycle, the profile of the host’s immune response and disease outcome remains unclear, and for several years it has been under intensive discussion with contradictory reports being published. Based on experimental studies, high iron was suggested to be an element of antiviral defense, potentially limiting HCV replication in CHC [17]. On the contrary, presence of iron overload markers is connected with resistance to IFN therapy and many studies suggest that iron depletion through phlebotomies is an effective strategy of improving liver status and IFN treatment efficacy in CHC patients [13, 15]. Also the genesis and individual predisposition to development of iron overload condition in CHC are still unknown. *HFE* gene mutations appeared to be the main causative agent of inherited iron overload among Caucasians, defined as hereditary hemochromatosis. Although the presence of mutant C282Y allele in HFE protein is known to be associated with elevated serum markers of iron metabolism and iron tissue accumulation in CHC, *HFE* mutations cannot be considered a major factor leading to iron overload in HCV-infected subjects [16]. Hepcidin, a 25-aa peptide hormone engaged in the control of body iron homeostasis, which is encoded by a gene located on chromosome 19q13, 13.8 kbp upstream from *IFNL3* sequence, was suggested to have a pivotal role in the development of iron overload syndrome in CHC [14, 18]. Hepcidin gene (*HAMP*) expression is regulated in response to changes in body iron as well as by inflammatory cytokines, hypoxia and growth signals [19]. Many reports show lower hepatic *HAMP* expression in CHC patients [14, 18, 20–22], and this downregulation may be due to HCV-induced oxidative stress which inhibits transcription factor C/EBPalpha [21].

Polymorphisms in *IFNL3*-*IFNL4* gene region, which impact the magnitude of cellular signaling in response to HCV infection, are known predictors of CHC disease course and treatment outcome. Also the role of markers of iron overload is discussed in the context of liver fibrosis progression, the efficacy of antiviral treatment and risk of carcinogenesis in CHC [12–16]. The precise molecular mechanisms linking *IFNL* SNPs and immune response to HCV infection, as well as the elusive role of iron overload in the pathogenesis of CHC, are not completely understood. This knowledge is important, as it could be used to establish new prognostic factors of HCV-related liver disease. Moreover, modulation of iron balance could be considered as a potential target for adjuvant therapy of HCV infection.

In this work, we investigated the association of *IFNL* polymorphisms in CHC patients with body iron indices, as well as with hepatic expression of selected genes involved in iron homeostasis and immune response to HCV infection. Among others, we measured the levels of three long non-coding RNAs (lncRNAs), whose expression is known to be modulated in response to viral infection and IFN treatment. One of these RNAs, lncRNA-CMPK2, called negative regulator of IFN response (NRIR), was previously significantly induced by IFN alpha (IFNA) and IFN gamma, but not by tumor necrosis factor alpha (TNFA) in various cell lines, as well as in HCV-infected human livers [23]. This lncRNA was shown to act through suppression of transcription of a subset of interferon-stimulated genes (ISGs) and to affect HCV replication. Another regulatory lncRNA, named a negative regulator of antiviral response (NRAV), was found to be downregulated in response to infection with several viruses. NRAV interacts with ZONAB transcription factor and inhibits expression of several antiviral effectors of innate immunity such as MxA or IFITM3 [24]. Expression of *BST2* IFN-stimulated positive regulator (BISPR) is upregulated in livers of HCV-infected patients, and in cells treated with IFNA2 or IFNL, but not with TNFA, and positively regulates expression of *BST2*, which encodes antiviral protein, tetherin [25].

In this report for the first time, we show an association of *IFNL* genotypes, indices of systemic iron balance, as well as hepatic expression of *HAMP* and an IFN-responsive lncRNA, NRIR, in CHC patients. Our results point to a specific regulatory pathway, which may be responsible for the impact of *IFNL* genotypes on the outcome of HCV infection, and underline the significance of immune response in development of iron overload in CHC.

## Methods

### Patient selection

Two hundred and twenty-two consecutive Polish patients (Caucasian origin) with diagnosis of CHC were included in this study. All patients met criteria for inclusion to treatment with pegylated IFN alpha (PEG-IFNA) and ribavirin in the Department of Infectious Diseases, Medical University of Gdansk. Exclusion criteria included: history of drug or alcohol abuse (>25 g alcohol intake/daily), diagnosis of chronic liver diseases other than HCV-related, coinfections HCV/HBV, HCV/HIV. HCV infection was detected and HCV genotyping was performed as described previously [22]. In recruited patients, liver function tests: activity of alanine and aspartate aminotransferases (ALT, AST), gamma-glutamyl transpeptidase (GGT), serum bilirubin concentration, blood morphology and serum iron content markers: iron and ferritin concentration, and transferrin saturation were performed. Normal upper reference values for serum concentration of iron were >140 ug/dl, for transferrin saturation 50 % in men; 45 % in women and for serum ferritin concentration, >300 ng/ml in men; >200 ng/ml in women [26].

From the initially selected group of 222 subjects, we excluded 20 patients for whom we identified at least one mutant *HFE* C282Y (rs1800562) allele to exclude its possible impact on iron overload development. The liver oligobiopsy was done in 185/192 CHC patients. In seven CHC patients, liver biopsy was not carried out because of contraindications. The preparation of liver specimen and classification of inflammation activity, fibrosis and liver iron deposits were previously described [27]. In the studied cohort, liver iron deposits were assessed only in hepatocytes and not in Kupffer cells. Assessment of hepatocyte steatosis was done on a scale 0–3 referring to amounts of hepatocyte surface area involved by steatosis (0 = <5 %; 1 = 5–33 %; 2 = >33–66 %; 3 = >66 %). SVR was defined as undetectable HCV RNA 24 weeks after completing PEG-IFNA and ribavirin treatment.

The study protocol was approved by the Local Independent Bioethics Committee at the Medical University of Gdansk (NKEB 246/2011) in compliance with the Declaration of Helsinki. All enrolled participants of this study provided written informed consent.

### SNP genotyping

Genomic DNA was isolated from whole blood samples using QIAamp DNA Blood Mini Kit (Qiagen, Germany) according to manufacturer’s instructions. Genotyping of rs1800562 (C282Y *HFE*) was performed with MassArray^®^ mass spectrometer (Agena, US) using IPLEX^®^Gold Complete genotyping set with SpectroCHIP^®^ II (Agena, US) as will be described elsewhere (Woziwodzka A, 2016, unpublished data). Genotyping of four SNPs: rs368234815, rs12979860, rs8099917 and rs12980275, was performed using allele-specific PCR as described in Supporting information.

### Gene expression analysis

Total RNA from biopsy liver tissue from 105 CHC patients was isolated using RNeasy Mini Kit (Qiagen, Germany). The cDNA was synthesized with QuantiTect Reverse Transcription Kit (Qiagen, Germany) from 250 ng of total RNA. All procedures were carried out according to the manufacturer’s instructions. qRT-PCR amplifications were performed in triplicates using LightCycler 480 system (Roche Applied Science, Germany). We have analyzed expression of genes associated with iron metabolism (*HAMP*, *FPN1*), inflammation (*TNFA*), and IFN response (*RSAD2*, interferon lambda receptor 1, *IFNLR1*, NRIR, BISPR, NRAV). Primers are listed in Table S2, and cycling conditions are described in Supporting information.

### Statistical analysis

Statistical analysis was carried out using data analysis software STATISTICA version 10 (StatSoft, Inc., USA). All statistical data were presented as a mean ± standard error (SE) or median value (histopathological data). SE was used since the distributions of data were skewed. The analysis of differences between variations was performed using nonparametric statistics: the Mann–Whitney *U* test, the Chi-square test, Yates’ Chi-square test and Spearman’s rank-order correlation coefficient test. The Bonferroni correction was applied in multiple testing procedures. Multiple logistic regression models were adjusted for age and sex. LD of analyzed SNPs was evaluated using MIDAS software [28]. All statistical tests were two-tailed. *P* values <0.05 were considered statistically significant.

## Results

Characteristics of patients enrolled in the study are shown in Table S3. Serum iron, transferrin saturation and ferritin positively correlated with age, ALT, GGT, bilirubin and hemoglobin concentration as well as fibrosis stage and degree of hepatocyte iron deposits in liver biopsy samples (Table S4). Additionally, serum iron, transferrin saturation and ferritin as well as the degree of iron deposition in the liver were all higher in men (*P* value 0.002, 0.002, <0.000,001 and 0.0003, respectively). Patients achieving SVR had significantly lower serum iron levels (*P* = 0.003). No other significant correlations of therapeutic outcome were found (results not shown).

Genotypes for four SNPs were obtained for all 192 patients included in the study. Rs368234815 ΔG/T and rs12979860 T/C, both within *IFNL4* gene region, were in a complete LD (r^2^ = 1), and therefore further in the text we will only refer to rs12979860. Minor allele frequencies for rs12979860 (T), rs8099917 (G) and rs12980275 (G) were 0.46, 0.3 and 0.43, respectively. The SNPs rs12979860 and rs12980275 were in a strong LD (r^2^ = 0.87), while rs12979860 and rs8099917 as well as rs12980275 and rs8099917 were independent (r^2^ = 0.43 and r^2^ = 0.46, respectively).

**Fig. 1:**
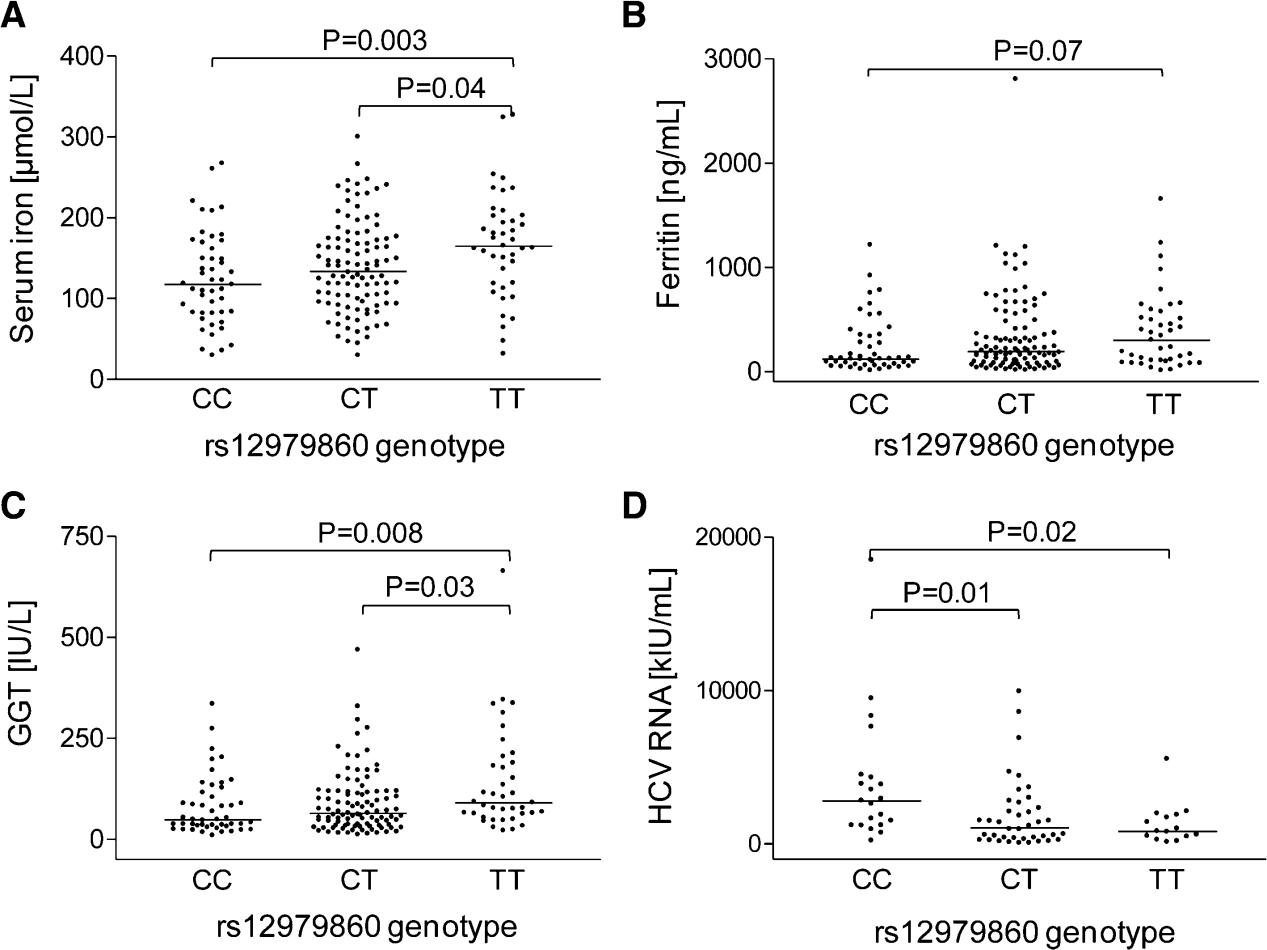
Association of rs12979860 with biochemical parameters. Serum iron (**a**), ferritin (**b**), GGT (**c**) and HCV load (**d**) were measured in samples from CHC patients. For serum iron, ferritin and GGT *n*(CC) = 50, *n*(CT) = 103, *n*(TT) = 39; for viral load *n*(CC) = 24, *n*(CT) = 43, *n*(TT) = 16. *Each dot* represents a data point for one patient

Biochemical and histopathological characteristics of patients with rs12979860 are shown in Table 1 and Fig. 1, while the data for SNPs rs8099917 and rs12980275 can be found in Table S5. Homozygosity in any of the major alleles in tested SNPs associates with lower GGT activity, with lower serum iron and with an elevated viral load. Patients bearing rs12979860 CC have lower serum ferritin and hemoglobin concentration and show an increased degree of inflammation in the liver tissue. On the other hand, rs12979860 TT or rs8099917 GG genotypes associate with an increased degree of steatosis. Logistic regression analysis showed that homozygosity in any of the unfavorable alleles in the studied SNPs is connected with an abnormally high serum iron concentration, while rs8099917 GG and rs12980275 GG independently associate with serum ferritin level above normal (Table 2). At the same time, elevated serum iron is less prevalent among patients bearing at least one favorable allele in each of tested loci (Table  2).

**Table 1:** Selected characteristics of HCV-infected patients with rs12979860

Variables	rs12979860 genotype					
	CC (*n* = 50)	TT + CT (*n* = 142)	*P*	TT (*n* = 39)	CC + CT (*n* = 153)	*P*
Gender (M/F)	27/23	90/52	0.23	23/16	94/59	0.78
Age (years)	46 ± 2	47 ± 1	0.81	48 ± 2	47 ± 1	0.51
ALT (IU/L)	109 ± 12	99 ± 6	0.60	112 ± 13	99 ± 6	0.24
GGT (IU/L)	79 ± 10	108 ± 8	0.06	144 ± 21	85 ± 6	**0.001**
Hemoglobin (g/dL)	14 ± 0.3	15 ± 0.1	**0.006**	15 ± 0.2	15 ± 0.1	0.18
Iron (μg/dL)	124 ± 8	147 ± 5	**0.02**	162 ± 10	136 ± 5	**0.008**
Transferrin saturation (%)	39 ± 3	40 ± 2	0.46	40 ± 3	40 ± 2	0.43
Ferritin (ng/mL)	238 ± 37	345 ± 32	**0.04**	379 ± 59	301 ± 29	0.17

Histopathology	CC (*n* = 48)	TT + CT (*n* = 137)	*P*	TT (*n* = 39)	CC + CT (*n* = 146)	*P*
Hepatocyte steatosis present	31 (65 %)	81 (59 %)	0.73	26 (67 %)	86 (59 %)	0.59
Hepatocyte iron deposits present	14 (29 %)	47 (34 %)	0.59	11 (28 %)	50 (34 %)	0.45
Inflammation	2 (1/3)^a^	2 (1/2)^a^	**0.03**	2	2	0.68
Fibrosis	2	2	0.83	2	2	0.53
Iron deposits	0	0	0.63	0	0	0.73
Steatosis	1	1	0.40	2	1	**0.03**

	CC (*n* = 24)	TT + CT (*n* = 59)	*P*	TT (*n* = 16)	CC + CT (*n* = 67)	*P*
Viral load (kIU/mL)	3843 ± 815	1678 ± 271	**0.0009**	1212 ± 334	2565 ± 383	0.13

*P* values for statistically significant differences between groups are shown in bold

Data analyzed in a dominant model for patients homozygous in major C or minor T allele. Quantitative biochemical data are shown as mean ± SE; data for inflammation, fibrosis, iron deposits and steatosis are shown as median values

^a^Percentiles (25th/75th)

**Table 2:** Association between *IFNL* genotypes and abnormally elevated serum iron indices

*IFNL* genotype	Total *n*	Serum iron above normal			Serum ferritin above normal		
		*n* (%)	OR (95 % CI)	*P*	*n* (%)	OR (95 % CI)	*P*
rs12979860							
TT	39	28 (72)	3.0 (1.4–6.6)	**0.005**	20 (51)	1.5 (0.7–3.3)	0.27
CC	50	17 (34)	0.5 (0.2–1.0)	**0.047**	15 (30)	0.7 (0.3–1.5)	0.33
rs8099917							
GG	19	15 (79)	4.5 (1.4–14.4)	**0.01**	13 (68)	4.1 (1.4–12.3)	**0.01**
TT	95	42 (44)	0.7 (0.4–1.3)	0.33	35 (37)	1.0 (0.5–1.8)	0.91
rs12980275							
GG	33	22 (67)	2.4 (1.1– 5.5)	**0.03**	18 (54)	2.3 (1.0–5.2)	**0.049**
AA	56	20 (36)	0.5 (0.2– 1.0)	0.06	18 (32)	0.8 (0.4–1.7)	0.58
rs12979860, rs8099917, rs12980275							
T, G, G	95	52 (55)	1.4 (0.8–2.6)	0.21	39 (41)	1.0 (0.5–1.9)	0.98
C, T, A	151	65 (43)	0.3 (0.1–0.7)	**0.003**	55 (36)	0.6 (0.3–1.3)	0.18
TT, GG, GG	17	13 (76)	3.9 (1.2–13.0)	**0.02**	12 (71)	4.9 (1.5–16.1)	**0.008**
CC, TT, AA	49	17 (35)	0.5 (0.3–1.1)	0.08	14 (29)	0.7 (0.3–1.4)	0.30

*P* values for statistically significant differences between groups are shown in bold

Results of multivariate logistic regression analyses, adjusted for age and sex

*OR* odds ratio, *CI* confidence intervals

Body iron indices associate with hepatic expression of *HAMP* and immune response genes (Table S7). Liver *HAMP* expression correlated with serum iron indices as well as with the degree of hepatocyte iron deposition in patients biopsy samples (Fig. S1), and it was significantly higher in male subjects (*P* 0.004). Iron indices were independent of ferroportin (*FPN1*) expression. The mRNA level of *TNFA* correlated with the degree of histopathological alterations in liver tissue (Table S7). Both *RSAD2* and NRIR expression significantly associated with ferritin and GGT concentration, while other lncRNAs, BISPR and NRAV, linked with serum iron level (Table S7). Expression of *FPN1, IFNLR1*, BISPR and NRAV strongly correlated together, while *RSAD2* associated the most with NRIR level (Table S6).

**Fig. 2:**
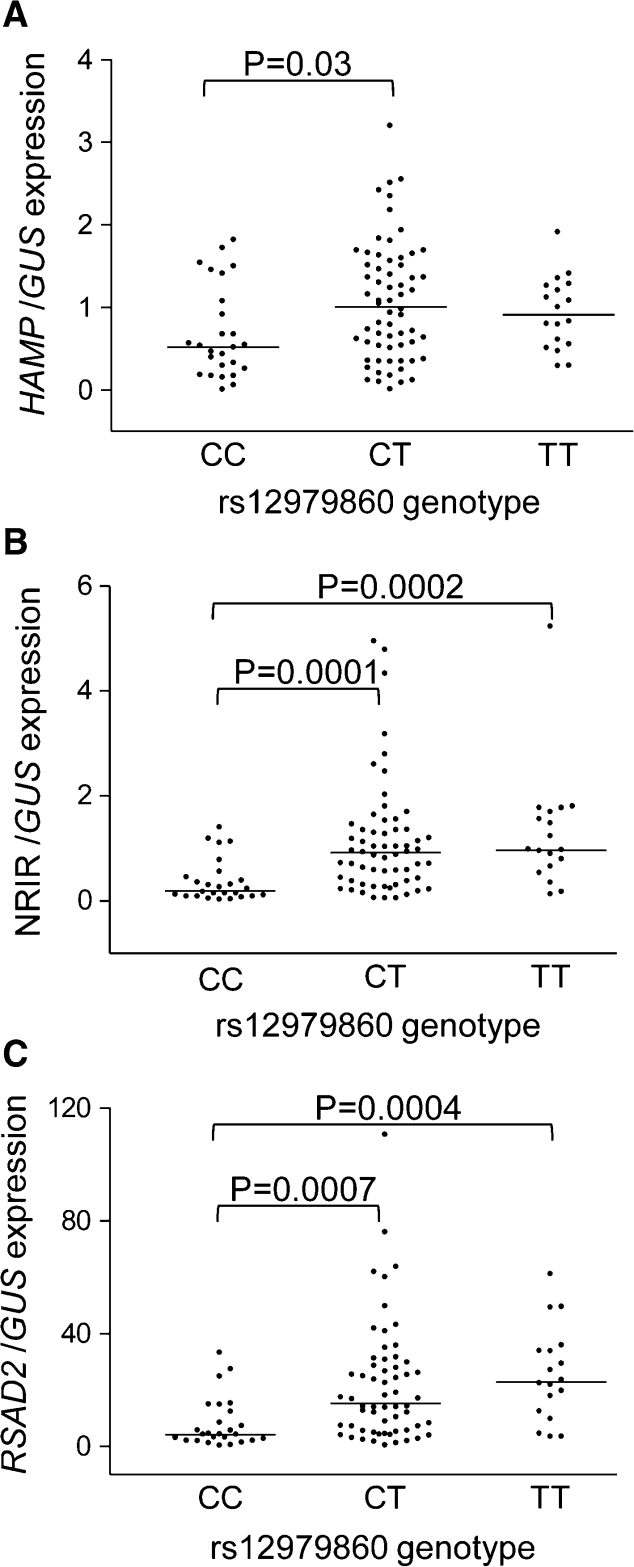
Association of rs12979860 with hepatic gene expression. Expression of *HAMP* (**a**), NRIR (**b**) and *RSAD2* (**c**) was measured in 105 liver biopsy samples of CHC patients; *n*(CC) = 26, *n*(CT) = 61 and *n*(TT) = 18. *Each dot* represents a data point for one patient

Lower *HAMP* mRNA level was observed in patients with a favorable rs12979860 CC genotype (Fig. 2a). No significant differences in *HAMP* expression were found for two other SNPs. All *IFNL* polymorphisms strongly associated with hepatic expression of one of IFN-responsive lncRNAs, NRIR, and *RSAD2* gene (Fig. 2b, c; Fig. S2), but not *IFNLR1*, BISPR or NRAV (Fig. S3).

## Discussion

### IFNL genotypes associate with body iron indices in CHC

In this work, we demonstrate that *IFNL* polymorphisms associate with the presence of serum markers of iron overload in CHC patients. Additionally, genotypes in *IFNL* region unfavorable for disease outcome and markers of serum and hepatocyte iron overload are both associated with higher GGT activity levels and liver steatosis. Increased lipid accumulation in the liver as well as elevated activity of GGT belongs to the group of strong negative prognostic factors of progressive liver damage [29, 30]. The reciprocal interaction between immune system and body iron level has been well documented [28, 31]. Deficiency in proteins of the adaptive immune response such as β-microglobulin or MHC class I leads to iron overload in mice [32]. In fact, in human macrophages the expression of iron homeostasis genes, encoding ferritin and ferroportin, is modulated by inflammatory cytokines [33]. In CHC patients, the beginning of PEG-IFNA therapy is associated with a decrease in systemic iron level. The intensity of this decline correlates with the response to treatment, and hypoferremia following first doses of IFN is a good indicator of the drug efficacy [34]. Long-term observation of patients undergoing frequent transfusions who develop systemic iron overload brings evidence for impaired function of the immune system. The resulting increase in the susceptibility to infection can be diminished with iron-chelating therapy [35]. Excess of body iron decreases the ratio of Treg to helper Th17 cells [36], which may facilitate establishing of chronic HCV infection [37].

### HAMP expression associates with IFNL genotype and iron indices

Hepatic *HAMP* expression positively correlated with serum iron indices and with the presence of hepatocyte iron deposits in the liver. This contradicts earlier suggestions that systemic iron overload observed in CHC patients may result from downregulation of *HAMP* expression [18, 20]. In our study, the presence of favorable *IFNL* rs12979860 CC genotype in CHC patients correlated with lower *HAMP* expression. At the same time, hepatic expression of *FPN1* was linked neither with *IFNL* genotype nor with iron indices. Other authors have found that a decreased *HAMP* and *FPN1* expression in the liver of CHC patients before the start of IFN and RBV therapy correlated with SVR after the treatment in patients of Japanese origin [38]. Hepcidin synthesis is induced by IFNA through the Jak/STAT3 signaling pathway, and increased serum hepcidin was observed in HCV patients following a single dose of PEG-IFNA. The resulting systemic iron withdrawal was the most pronounced one in those with the strongest viral response to PEG-IFNA [34]. Successful therapy with PEG-IFNA was also accompanied with an elevation of serum hepcidin together with a decrease in serum iron and ferritin concentration [39]. Taken together, these data suggest that the magnitude of IFN-induced inflammation impacts hepatic *HAMP* expression of CHC patients, independently of body iron stores.

### IFNL polymorphisms associate with immune responsiveness to HCV

The molecular mechanisms linking *IFNL* SNPs with immune response to HCV infection and therapy are not fully understood. Minor *IFNL* alleles are connected with a pre-activated state of immune system in CHC, characterized by an increased expression levels of IFN-stimulated genes (ISGs), as well as the presence of IFNL4 in liver tissue [10, 40]. In CHC patients bearing *IFNL* rs12979860 TT genotype, stimulation with IFNA results in an increased responsiveness of NK cells [41] and stronger induction of hepatic expression of IFNL receptor, *IFNLR1* [6]. It was earlier shown that *IFNL* genotype and hepatic expression of four ISGs: *IFI27*, *ISG15*, *RSAD2* and *HTATIP2,* are independent predictors of response to treatment with IFNA and ribavirin [42]. In our work, we could not find a statistically significant association of therapeutic outcome with studied genotypes or hepatic gene expression. This is probably due to a very limited data available, as only 74 patients completed the whole cycle of PEG-IFNA–ribavirin therapy (Table S3). We have, however, confirmed a strong association of *IFNL* polymorphisms with the hepatic expression of two ISGs, *RSAD2,* encoding antiviral protein, viperin and lncRNA NRIR.

The impact of *IFNL* genotype on the magnitude of immune response to HCV infection changes the dynamics of viral clearance. In our study, rs12979860 CC-bearing patients showed an increased necroinflammatory activity in liver tissue and higher baseline viral load. In a meta-analysis study performed by Sato et al. [9], the favorable *IFNL* genotypes (rs8099917 and rs12979860) were associated with a higher necroinflammatory activity and increased possibility of fibrosis in the liver. The presence of a favorable rs12979860 CC genotype was connected with elevated baseline viral load followed by a more rapid decline in HCV counts after 28 days of treatment and a better response to therapy [43, 44].

In order to gain more insight into mechanism of the role of *IFNL* polymorphisms in CHC, we analyzed hepatic expression of three lncRNAs, which are known to regulate cellular IFN response. We found a significant correlation between hepatic expression of NRIR, but not BISPR or NRAV, and all tested *IFNL* genotypes, with the strongest association for rs12979860/rs368234815. NRIR expression was also differently related to iron biochemical indices than BISPR and NRAV. The reason for this selective association is currently unknown. Expression of NRIR and BISPR, but not NRAV, was previously shown to be regulated through the JAK/STAT2 pathway [23, 24, 45]. Additionally, these lncRNAs exhibit distinct kinetics of induction or downregulation in response to different types of IFN [23–25]. It can be hypothesized that in CHC hepatic NRIR expression is induced by IFNL4 and that the negative impact of this lncRNA on immune response to IFN might be responsible for the therapeutic failure in rs12979860 TT patients.

In conclusion, this report shows a link between body iron balance and immune response to HCV infection, and points to a specific molecular pathway, involving NRIR, and linking *IFNL* polymorphisms and IFN response. The pre-activation of inflammatory response in CHC patients homozygous in minor *IFNL* rs12979860 T allele leads to exacerbation of liver damage and makes immune system refractory to therapeutic stimulation. Dysregulation of iron balance associated with this genotype can further impair immune response and facilitate disease progression. We hypothesize that the overly activated IFN signaling, evidenced by increased hepatic ISGs expression (*RSAD2* and NRIR), which is associated with unfavorable *IFNL* genotype, may be one of the factors contributing to elevated iron indices observed in CHC patients. To verify this, a further research with a larger cohort of patients including data on treatment outcome is needed. Also future studies on the role and molecular function of NRIR will provide more insight in the association of *IFNL* genotypes, IFN response and iron homeostasis in CHC.

## Electronic supplementary material

Below is the link to the electronic supplementary material.

Supplementary material 1 (DOCX 125 kb)

## References

[1] Wedemeyer H, Duberg AS, Buti M (2014). Strategies to manage hepatitis C virus (HCV) disease burden. J Viral Hepat.

[2] Ge D, Fellay J, Thompson AJ (2009). Genetic variation in IL28B predicts hepatitis C treatment-induced viral clearance. Nature.

[3] Suppiah V, Moldovan M, Ahlenstiel G (2009). IL28B is associated with response to chronic hepatitis C interferon-alpha and ribavirin therapy. Nat Genet.

[4] Tanaka Y, Nishida N, Sugiyama M (2009). Genome-wide association of IL28B with response to pegylated interferon-alpha and ribavirin therapy for chronic hepatitis C. Nat Genet.

[5] Urban TJ, Thompson AJ, Bradrick SS (2010). IL28B genotype is associated with differential expression of intrahepatic interferon-stimulated genes in patients with chronic hepatitis C. Hepatology.

[6] Duong FH, Trincucci G, Boldanova T (2014). IFN-λ receptor 1 expression is induced in chronic hepatitis C and correlates with the IFN-λ3 genotype and with nonresponsiveness to IFN-α therapies. J Exp Med.

[7] Tillmann HL, Patel K, Muir AJ (2011). Beneficial IL28B genotype associated with lower frequency of hepatic steatosis in patients with chronic hepatitis C. J Hepatol.

[8] Rojas Á, del Campo JA, Maraver M (2014). Hepatitis C virus infection alters lipid metabolism depending on IL28B polymorphism and viral genotype and modulates gene expression in vivo and in vitro. J Viral Hepat.

[9] Sato M, Kondo M, Tateishi R (2014). Impact of IL28B genetic variation on HCV-induced liver fibrosis, inflammation, and steatosis: a meta-analysis. PLoS One.

[10] Amanzada A, Kopp W, Spengler U, Ramadori G, Mihm S (2013). Interferon-λ4 (IFNL4) transcript expression in human liver tissue samples. PLoS One.

[11] Prokunina-Olsson L, Muchmore B, Tang W (2013). A variant upstream of IFNL3 (IL28B) creating a novel interferon gene IFNL4 is associated with impaired clearance of hepatitis C virus. Nat Genet.

[12] Bonkovsky HL, Naishadham D, Lambrecht RW (2006). Roles of iron and HFE mutations on severity and response to therapy during retreatment of advanced chronic hepatitis C. Gastroenterology.

[13] Di Bisceglie AM, Bonkovsky HL, Chopra S (2000). Iron reduction as an adjuvant to interferon therapy in patients with chronic hepatitis C who have previously not responded to interferon: a multicenter, prospective, randomized, controlled trial. Hepatology.

[14] Fujita N, Horiike S, Sugimoto R (2007). Hepatic oxidative DNA damage correlates with iron overload in chronic hepatitis C patients. Free Radic Biol Med.

[15] Price L, Kowdley KV (2009). The role of iron in the pathophysiology and treatment of chronic hepatitis C. Can J Gastroenterol..

[16] Sikorska K, Stalke P, Izycka-Swieszewska E, Romanowski T, Bielawski KP (2010). The role of iron overload and HFE gene mutations in the era of pegylated interferon and ribavirin treatment of chronic hepatitis C. Med Sci Monit..

[17] Fillebeen C, Pantopoulos K (2010). Iron inhibits replication of infectious hepatitis C virus in permissive Huh7.5.1 cells. J Hepatol.

[18] Girelli D, Pasino M, Goodnough JB (2009). Reduced serum hepcidin levels in patients with chronic hepatitis C. J Hepatol.

[19] Nicolas G, Chauvet C, Viatte L (2002). The gene encoding the iron regulatory peptide hepcidin is regulated by anemia, hypoxia, and inflammation. J Clin Invest.

[20] Fujita N, Sugimoto R, Urawa N (2007). Influence of phlebotomy on iron-related gene expression levels in the livers of patients with chronic hepatitis C. J Gastroenterol.

[21] Miura K, Taura K, Kodama Y, Schnabl B, Brenner DA (2008). Hepatitis C virus-induced oxidative stress suppresses hepcidin expression through increased histone deacetylase activity. Hepatology.

[22] Sikorska K, Romanowski T, Stalke P, Izycka Swieszewska E, Bielawski KP (2014). Association of hepcidin mRNA expression with hepatocyte iron accumulation and effects of antiviral therapy in chronic hepatitis C infection. Hepat Mon.

[23] Kambara H, Niazi F, Kostadinova L (2014). Negative regulation of the interferon response by an interferon-induced long non-coding RNA. Nucl Acids Res.

[24] Ouyang J, Zhu X, Chen Y (2014). NRAV, a long noncoding RNA, modulates antiviral responses through suppression of interferon-stimulated gene transcription. Cell Host Microbe.

[25] Barriocanal M, Carnero E, Segura V, Fortes P (2014). Long non-coding RNA BST2/BISPR is induced by IFN and regulates the expression of the antiviral factor tetherin. Front Immunol.

[26] Beaton MD, Adams PC (2012). Treatment of hyperferritinemia. Ann Hepatol.

[27] Sikorska K, Bielawski KP, Stalke P (2005). HFE gene mutations in Polish patients with disturbances of iron metabolism: an initial assessment. Int J Mol Med.

[28] Gaunt TR, Rodriguez S, Zapata C, Day IN (2006). MIDAS: software for analysis and visualisation of interallelic disequilibrium between multiallelic markers. BMC Bioinform.

[29] Lange CM, Kutalik Z, Morikawa K (2012). Serum ferritin levels are associated with a distinct phenotype of chronic hepatitis C poorly responding to pegylated interferon-alpha and ribavirin therapy. Hepatology.

[30] Everhart JE, Wright EC (2013). Association of γ-glutamyltransferase (GGT) activity with treatment and clinical outcomes in chronic hepatitis C (HCV). Hepatology.

[31] Cherayil BJ (2010). Iron and immunity: immunological consequences of iron deficiency and overload. Arch Immunol Ther Exp (Warsz).

[32] Porto G, De Sousa M (2007). Iron overload and immunity. World J Gastroenterol.

[33] Recalcati S, Locati M, Marini A (2010). Differential regulation of iron homeostasis during human macrophage polarized activation. Eur J Immunol.

[34] Ryan JD, Altamura S, Devitt E (2012). Pegylated interferon-alpha induced hypoferremia is associated with the immediate response to treatment in hepatitis C. Hepatology.

[35] Cunningham-Rundles S, Giardina PJ, Grady RW, Califano C, McKenzie P, De Sousa M (2000). Effect of transfusional iron overload on immune response. J Infect Dis.

[36] Ezoe S, Yokota T, Ishibashi T, Oritani K, Kanakura Y (2013). Iron overload effects on immune system through the cytokine secretion by macrophage. Blood.

[37] Hao C, Zhou Y, He Y (2014). Imbalance of regulatory T cells and T helper type 17 cells in patients with chronic hepatitis C. Immunology.

[38] Kohjima M, Yoshimoto T, Enjoji M (2015). Hepcidin/ferroportin expression levels involve efficacy of pegylated-interferon plus ribavirin in hepatitis C virus-infected liver. World J Gastroenterol.

[39] Fujita N, Sugimoto R, Motonishi S (2008). Patients with chronic hepatitis C achieving a sustained virological response to peginterferon and ribavirin therapy recover from impaired hepcidin secretion. J Hepatol.

[40] Pfeffer LM, Li K, Fleckenstein JF (2014). An interferon response gene signature is associated with the therapeutic response of hepatitis c patients. Shoukry NH, ed. PLoS One.

[41] Rogalska-Taranta M, Markova AA, Taranta A (2015). Altered effector functions of NK cells in chronic hepatitis C are associated with IFNL3 polymorphism. J Leukoc Biol.

[42] Dill MT, Duong FH, Vogt JE (2011). Interferon-induced gene expression is a stronger predictor of treatment response than IL28B genotype in patients with hepatitis C. Gastroenterology.

[43] Howell CD, Gorden A, Ryan KA (2012). Single nucleotide polymorphism upstream of interleukin 28B associated with phase 1 and phase 2 of early viral kinetics in patients infected with HCV genotype 1. J Hepatol.

[44] Naggie S, Osinusi A, Katsounas A (2012). Dysregulation of innate immunity in hepatitis C virus genotype 1 IL28B-unfavorable genotype patients: impaired viral kinetics and therapeutic response. Hepatology.

[45] Kambara H, Gunawardane L, Zebrowski E (2014). Regulation of Interferon-Stimulated Gene BST2 by a lncRNA Transcribed from a Shared Bidirectional Promoter. Front Immunol.

