# Supplementary material for: Interferon lambda polymorphisms associate with body iron indices and hepatic expression of interferon-responsive long non-coding RNA in chronic hepatitis C
Source: Clin Exp Med. 2016 Apr 28;17(2):225–32. doi: 10.1007/s10238-016-0423-4 (PMC5403869; doi:10.1007/s10238-016-0423-4)
Supplement: Supplementary file 1 — Supplementary material 1 (DOCX 125 kb) [file 10238_2016_423_MOESM1_ESM.docx]

Clinical and Experimental Medicine

Interferon lambda polymorphisms associate with body iron indices and hepatic expression of interferon responsive long non-coding RNA in chronic hepatitis C

Anna Wróblewska, Agnieszka Bernat, Anna Woziwodzka, Joanna Markiewicz, Tomasz Romanowski, Krzysztof P. Bielawski, Tomasz Smiatacz, Katarzyna Sikorska

**Correspondence:**

Katarzyna Sikorska MD, PhD

Department of Tropical Medicine and Epidemiology, Medical University of Gdansk, Poland, Powstania Styczniowego 9b, 81-519, Gdynia

Tel./fax: +48 58 349 17 60; Email: ksikorska@gumed.edu.pl

**Table of contents**

**Patients and methods ....................................................................................................3**

SNP genotyping. Table S1. Primers used for SNP genotyping with AS-PCR.................3

Quantitative gene expression analysis...............................................................................4

Table S3. Characteristics of patients enrolled in the study................................................5

**Results...............................................................................................................................6**

Table S4. Association of serum iron indices and iron deposits with other biochemical and histopathological parameters......................................................................6

Table S5. Selected characteristics of HCV infected patients with rs8099917 and rs12980275 *IFNL* polymorphisms....................................................................7

Table S6. Correlation between hepatic gene expression...................................................8

Table S7. Association of hepatic gene expression with biochemical and histopathological parameters.........................................................................................................9

Figure S1. *HAMP* expression and iron indices................................................................10

Figure S2. Association of rs8099917 and rs12980275 genotypes with hepatic expression of NRIR and *RSAD2*......................................................................................11

Figure S3. Rs12979860 genotype is not associated with hepatic expression of *IFNLR1*, BISPR and NRAV..........................................................................................12

**References.......................................................................................................................13SNP genotyping**

Genotyping of four SNPs within *IFNL3* and *IFNL4* gene region: rs12979860, rs368234815, rs8099917 and rs12980275 was performed using allele-specific PCR method with β-globin gene fragment as a positive control. Primers designed using a Web-based Allele-Specific PCR designing tool [1] are listed in Table S1.

**Table S1** Primers used for SNP genotyping with AS-PCR

| **Primer name** | **Primer sequence 5’-->3’** | **Product lenght** |
| --- | --- | --- |
| rs12979860_Common_F | CTAACCTCTGCACAGTCTGG | 121bp |
| rs12979860_C_R | GAGTGCAATTCAACCCTGGTTAG |  |
| rs12979860_T_R | GAGTGCAATTCAACCCTGGTTAA |  |
| rs368234815_Common_F | TCTGTGATTGACCCTGAGCC | 300bp |
| rs368234815_G_R | CTGTGCACGGTGATCGCAAC |  |
| rs368234815_T_R | CTGTGCACGGTGATCGCAAA |  |
| rs8099917_Common_R | CTAGCTCTTGTCATTTGCCT | 239bp |
| rs8099917_G_F | TTTCCTTTCTGTGAGCAACG |  |
| rs8099917_T_F | TTTCCTTTCTGTGAGCAACT |  |
| rs12980275_Common_F | AGCAAGAGGAGGGAAGGAAG | 176bp |
| rs12980275_A_R | CCGGCAAATATTTAGACACGTAT |  |
| rs12980275_G_R | CCGGCAAATATTTAGACACGTAC |  |
| B-globin_F | TTGGACCCAGAGGTTCTTTG | 122bp † |
| B-globin_1R | GAGCCAGGCCATCACTAAAG |  |
| B-globin_F | TTGGACCCAGAGGTTCTTTG | 199bp † |
| B-globin_2R | GTTCTCAGGATCCACGTGCA |  |

Mismatches at the second position from the 3’ end of the allele specific primers are underlined. †Amplification of short β-globin gene fragment (122bp) was used for genotyping of rs12980275 and rs8099917 while longer fragment (199bp) served as a control for genotyping of rs12979860.

PCR reactions consisted of 2mM MgCl2, 5 pmol of each dNTP, 10 pmol of each primer, 1 u Taq polymerase (Thermo Scientific), 4% DMSO (Sigma), 0.2 mg/mL BSA (Sigma) and 20-50 ng genomic DNA. Cycling conditions were 95°C 3 min. and 30 cycles of 30s 95°C, 30s annealing step and 30s 72°C. Annealing temperatures were the following: for rs12979860 58°C (allele C) and 60°C (allele T), for rs368234815 55°C (allele T) and 60°C (allele G), for rs8099917 - 50°C, and for rs12980275 - 56°C. For confirmation of the results random DNA samples were subjected to sequencing.

**Quantitative gene expression analysis**

Primer sequences for gene expression analysis are shown in Table S2. Primers designed within this study were generated using Primer-BLAST tool [2].

**Table S2** Primers used for quantitative gene expression analysis

| **Primer name** | **Primer sequence 5’-->3’** | **Reference** |
| --- | --- | --- |
| GUS_F | CGAGAGTGCTGGGGAATAAA | this study |
| GUS_R | CCTGGTTTCATTGGCAATCT |  |
| HAMP_F | AGACACCCACTTCCCCATCT | this study |
| HAMP_R | CACATCCCACACTTTGATCG |  |
| FPN1_F | CAGGGACTGAGTGGTTCCAT | this study |
| FPN1_R | ACCACATTTTCGACGTAGCC |  |
| RSAD2_F | CTTTGTGCTGCCCCTTGAG | this study |
| RSAD2_R | CACCAACTTGCCCAGGTATT |  |
| TNFA_F | TCCTTCAGACACCCTCAACC | this study |
| TNFA_R | AGGCCCCAGTTTGAATTCTT |  |
| IFNLR1_F | CAGTGTCCCGAAATACAGCA | [3] |
| IFNLR1_R | tgtgtccagaaaagtccagggc |  |
| NRIR_F | TCACGATGCATGGGAAGACTA | this study |
| NRIR_R | AAGGAGGTTAGAGGTGTCTGC |  |
| BISPR_F | CGTGCCAGCCTGTATTCAT | [4] |
| BISPR_R | CCAGTCTCACCTGTTGCTCA |  |
| NRAV_F | CCCACATGGTAGATGGAACC | this study |
| NRAV_R | AGATCACGATGGCCAAGAAC |  |

The PCR mixture contained LightCycler 480 SYBR Green I Master (Roche Applied Science, Germany), 0.5 µM of forward and reverse primer and 1 µl of cDNA in a final volume of 10 µl. Amplification programme included 10s preincubation in 95°C and 45 cycles of 10s 95°C, 10s annealing at 60°C and 7s primer extension at 72°C. Melting curve analysis was performed after each run to exclude unspecific amplification. GUS was used as a reference gene for gene expression analysis as the most stably expressed housekeeping gene for human liver tissue [5].

**Table S3** Characteristics of patients enrolled in the study

| **Variable** | **Characteristics** |
| --- | --- |
| Gender (Male/Female) | 117/75 |
| Age (yr) | 19 - 77 (49) |
| Hemoglobin (g/dL) | 9.6 – 18.4 (14.9) |
| ALT (IU/L) | 14 – 431 (73) |
| AST (IU/L) | 3 - 369 (49) |
| GGT (IU/L) | 9 – 663 (68) |
| ALP (IU/L) | 25 - 272 (72) |
| Bilirubin (mg/dL) | 0.17 – 3.20 (0.69) |
| Iron (μg/dL) | 29 – 327 (140) |
| Transferrin saturation (%) | 6 - 100 (38) |
| Ferritin (ng/mL) | 6.5- 2800 (171) |
| HCV genotype (n=131) | 131 genotype 1  15 genotype 3  10 genotype 4 |
| HCV RNA (kIU/mL) (n=83) | 37.4 – 18500 (2304) |
| SVR/non-SVR | 26/48 |

Quantitative variables are presented as minimal - maximal values (median)

**Table S4** Association of serum iron indices and liver iron deposits with other biochemical and histopathological parameters

| **Variables** | **Serum parameters** | | | **Iron deposits** |
| --- | --- | --- | --- | --- |
|  | **Iron** | **Transferrin saturation** | **Ferritin** |  |
| Age | 0.1379 ^*^ | 0.1825 ^*^ | 0.2359 ^**^ | 0.1553 ^*^ |
| Hemoglobin | 0.3913 ^***^ | 0.3499 ^***^ | 0.3736 ^***^ | 0.1483 ^*^ |
| ALT | 0.5318 ^***^ | 0.4771 ^***^ | 0.5073 ^***^ | 0.2079 ^*^ |
| AST | 0.5099 ^***^ | 0.4447 ^***^ | 0.4603 ^***^ | 0.1869 ^*^ |
| GGT | 0.3878 ^***^ | 0.2833 ^***^ | 0.5397 ^***^ | 0.1978 ^*^ |
| Bilirubin | 0.3752 ^***^ | 0.4156 ^***^ | 0.3866 ^***^ | 0.1844 ^*^ |
| Iron | 1 | 0.8842 ^***^ | 0.6164 ^***^ | 0.3034 ^**^ |
| Transferrin saturation | 0.8842 ^***^ | 1 | 0.6351 ^***^ | 0.2506 ^*^ |
| Ferritin | 0.6164 ^***^ | 0.6351 ^***^ | 1 | 0.5061 ^***^ |
| **Histopathology** | | | | |
| Inflammation | 0.2003 ^*^ | NS | NS | NS |
| Fibrosis | 0.2357 ^**^ | 0.2366 ^*^ | 0.2489 ^**^ | NS |
| Iron deposits | 0.3035 ^**^ | 0.2506 ^*^ | 0.5061 ^***^ | 1 |
| Steatosis | 0.1819 ^*^ | 0.1794 ^*^ | NS | NS |

Spearman rank correlation coefficients; ^***^, p<0.000001; ^**^ p<0.001; ^*^, p<0.05; NS, not significant. For age and biochemical parameters n=192, for histopathological characteristics n=185.

**Table S5** Selected characteristics of HCV infected patients with rs8099917 and rs12980275 *IFNL* polymorphisms

|  | rs8099917 | | | | | | rs12980275 | | | | | |
| --- | --- | --- | --- | --- | --- | --- | --- | --- | --- | --- | --- | --- |
| Variables | TT  (n=95) | GG+GT  (n=97) | P | GG  (n=19) | TT+GT  (n=173) | P | AA  (n=56) | GG+AG  (n=136) | P | GG  (n=33) | AA+AG (n=159) | P |
| Gender (M/F) | 55/40 | 62/35 | 0.39 | 11/8 | 106/67 | 0.77 | 31/25 | 86/50 | 0.31 | 19/14 | 98/61 | 0.66 |
| Age (yr) | 46±1 | 47±1 | 0.50 | 49±2 | 46±1 | 0.26 | 45±2 | 47±1 | 0.42 | 48±3 | 46±1 | 0.32 |
| ALT (IU/L) | 106±9 | 97±7 | 0.41 | 115±18 | 100±6 | 0.18 | 105±11 | 100±7 | 0.66 | 111±13 | 99±6 | 0.23 |
| GGT (IU/L) | 85±9 | 108±10 | **0.01** | 187±37 | 87±6 | **0.0008** | 76±9 | 106±9 | **0.03** | 154±23 | 85±6 | **0.0004** |
| Hemoglobin (g/dL) | 15±0.2 | 15±0.2 | 0.28 | 15±0.3 | 14±0.1 | 0.49 | 14±0.3 | 15±0.1 | **0.03** | 15±0.3 | 15±0.1 | 0.48 |
| Iron (μg/dL) | 138±6 | 144±6 | 0.57 | 182±14 | 136±4 | **0.002** | 126±8 | 147±5 | **0.03** | 160±11 | 137±5 | **0.03** |
| Transferrin saturation (%) | 41±2 | 39±2 | 0.65 | 43±4 | 39±2 | 0.20 | 41±3 | 39 ±2 | 0.80 | 38±3 | 40±2 | 0.92 |
| Ferritin (ng/mL) | 307±40 | 327±33 | 0.29 | 508±100 | 296±26 | **0.02** | 240±34 | 349±33 | 0.05 | 402±68 | 299±28 | 0.18 |
| Histopathology | TT  (n=88) | GG+GT  (n=97) | P | GG  (n=17) | TT+GT (n=168) | P | AA  (n=52) | GG+AG  (n=133) | P | GG  (n=33) | AA+AG (n=152) | P |
| Hepatocyte steatosis present | 55 (63%) | 57 (59%) | 0.55 | 14 (82%) | 98 (58%) | 0.07 | 32 (62%) | 80 (60%) | 0.56 | 23 (70%) | 89 (59%) | 0.25 |
| Hepatocyte iron deposits present | 30 (34%) | 31 (32%) | 0.75 | 7 (41%) | 54 (32%) | 0.43 | 15 (29%) | 46 (35%) | 0.49 | 9 (27%) | 52 (34%) | 0.54 |
| Inflammation | 2 | 2 | 0.26 | 2 | 2 | 0.61 | 2 | 2 | 0.35 | 2 | 2 | 0.94 |
| Fibrosis | 2 | 2 | 0.93 | 2 | 2 | 0.75 | 2 | 2 | 0.91 | 2 | 2 | 0.76 |
| Iron deposits | 0 | 0 | 0.31 | 0 | 0 | 0.72 | 0 | 0 | 0.52 | 0 | 0 | 0.78 |
| Steatosis | 1 | 1 | 0.23 | 2 | 1 | **0.008** | 1 | 1 | 0.72 | 2 | 1 | 0.15 |
|  | TT  (n=41) | GG+GT  (n=42) | P | GG  (n=8) | TT+GT  (n=75) | P | AA  (n=26) | GG+AG  (n=57) | P | GG  (n=13) | AA+AG  (n=70) | P |
| Viral load (kIU/mL) | 3213±527 | 1373±304 | **5x10^-5^** | 799±264 | 2465±349 | 0.07 | 3659±764 | 1686±280 | **0.001** | 1231±403 | 2503±368 | 0.15 |

Data analyzed in a dominant model. Major alleles for rs8099917 and rs12980275 – T and A, respectively. Data for inflammation, fibrosis, iron deposits and steatosis are shown as median values.

|  | ***HAMP*** | ***FPN1*** | ***RSAD2*** | ***TNFA*** | ***IFNLR1*** | **NRIR** | **BISPR** | **NRAV** |
| --- | --- | --- | --- | --- | --- | --- | --- | --- |
| ***HAMP*** | 1 |  |  |  |  |  |  |  |
| ***FPN1*** | NS | 1 |  |  |  |  |  |  |
| ***RSAD2*** | NS | 0.520 ^***^ | 1 |  |  |  |  |  |
| ***TNFA*** | NS | 0.719 ^***^ | 0.425 ^**^ | 1 |  |  |  |  |
| ***IFNLR1*** | NS | **0.852** ^***^ | 0.650 ^***^ | 0.636 ^***^ | 1 |  |  |  |
| **NRIR** | NS | 0.423 ^**^ | **0.924** ^***^ | 0.321 ^*^ | 0.584 ^***^ | 1 |  |  |
| **BISPR** | NS | **0.860** ^***^ | 0.642 ^***^ | 0.677 ^***^ | **0.903** ^***^ | 0.551 ^***^ | 1 |  |
| **NRAV** | NS | **0.884** ^***^ | 0.514 ^***^ | 0.744 ^***^ | **0.825** ^***^ | 0.431 ^**^ | **0.859** ^***^ | 1 |

**Table S6** Correlation between hepatic gene expression

Spearman rank correlation coefficients, values above 0.8 are marked in bold; ^***^, p<0.000001; ^**^, p<0.00001; ^*^, p<0.001; NS, not significant. Gene expression in relation to GUS was measured in 105 liver biopsy samples from CHC patients

**Table S7** Association of hepatic gene expression with biochemical and histopathological parameters

| **Variable** | **Gene expression** | | | | | | | |
| --- | --- | --- | --- | --- | --- | --- | --- | --- |
|  | ***HAMP*** | ***FPN1*** | ***RSAD2*** | ***TNFA*** | ***IFNLR1*** | **NRIR** | **BISPR** | **NRAV** |
| Age | 0.198 ^*^ | NS | NS | 0.200 ^*^ | NS | NS | NS | NS |
| Hemoglobin | NS | NS | 0.214 ^*^ | NS | 0.220 ^*^ | 0.198 ^*^ | 0.229 ^*^ | 0.193 ^*^ |
| ALT | NS | NS | NS | 0.339 ^**^ | NS | NS | NS | NS |
| AST | NS | NS | NS | 0.404 ^*^ | NS | NS | NS | NS |
| GGT | NS | NS | 0.217 ^*^ | 0.210 ^*^ | NS | 0.219 ^c^ | NS | NS |
| Bilirubin | NS | NS | NS | NS | NS | NS | NS | NS |
| Iron | 0.277 ^*^ | NS | NS | 0.223 ^*^ | NS | NS | 0.208 ^*^ | 0.196 ^*^ |
| Transferrin saturation | 0.351 ^**^ | NS | NS | NS | NS | NS | NS | NS |
| Ferritin | 0.519 ^***^ | NS | 0.259 ^*^ | 0.292 ^*^ | NS | 0.304 ^*^ | 0.194 ^*^ | NS |
| **Histopathology** |  |  |  |  |  |  |  |  |
| Inflammation | NS | NS | NS | 0.283 ^*^ | NS | NS | NS | NS |
| Fibrosis | NS | NS | NS | 0.244 ^*^ | NS | NS | NS | NS |
| Iron deposits | 0.323 ^**^ | NS | NS | NS | NS | NS | NS | NS |
| Steatosis | NS | NS | NS | 0.234 ^*^ | NS | NS | NS | NS |

Spearman rank correlation coefficients; ^***^, p<0.000001; ^**^, p<0.0001; ^*^, p<0.05; NS, not significant. Gene expression in relation to GUS was measured in 105 liver biopsy samples from CHC patients

**Fig. S1** *HAMP* expression and iron indices.

*HAMP* expression in relation to the body iron indices: transferrin saturation (**a**), serum iron (**b**), ferritin (**c**), and iron deposits in the liver (**d**).

Gene expression was measured in 105 liver biopsy samples of CHC patients. Each square represents a data point for one patient. Dashed lines in (**a-c**) indicate predictive intervals (95%), r and P values for linear regression analysis are shown on each graph.

**Fig. S2** Association of rs8099917 and rs12980275 genotypes with hepatic expression of NRIR and *RSAD2*.

NRIR (**a**, **c**) and *RSAD2* (**b**, **d**) expression was measured in 105 liver biopsy samples from CHC patients. Number of patients: for rs8099917 (**a**-**b**) n(TT)=51, n(GT)=47, n(GG)=7, and for rs12980275 (C-D) n(AA)=30, n(AG)=58, n(GG)=17.

**Fig. S3** Rs12979860 genotype is not associated with hepatic expression of *IFNLR1*, BISPR and NRAV.

*IFNLR1* (**a**), BISPR (**b**) and NRAV (**c**) expression was measured in 105 liver biopsy samples from CHC patients. Number of patients: n(CC)=26, n(CT)=61, n(TT)=18. P values are from Kruskal-Wallis test.

**References**

1. Wangkumhang P, Chaichoompu K, Ngamphiw C, et al. WASP: a Web-based Allele-Specific PCR assay designing tool for detecting SNPs and mutations. BMC Genomics. 2007;8:275.

2. Ye J, Coulouris G, Zaretskaya I, Cutcutache I, Rozen S, Madden T. Primer-BLAST: A tool to design target-specific primers for polymerase chain reaction. BMC Bioinformatics. 2012;13:134.

3. Duong FH, Trincucci G, Boldanova T, et al. IFN-λ receptor 1 expression is induced in chronic hepatitis C and correlates with the IFN-λ3 genotype and with nonresponsiveness to IFN-α therapies. J Exp Med. 2014;211(5):857-68.

4. Barriocanal M, Carnero E, Segura V, Fortes P. Long Non-Coding RNA BST2/BISPR is Induced by IFN and Regulates the Expression of the Antiviral Factor Tetherin. Front Immunol. 2014;5:655.

5. Romanowski T, Sikorska K, Bielawski KP. GUS and PMM1 as suitable reference genes for gene expression analysis in the liver tissue of patients with chronic hepatitis. Med Sci Monit. 2008;14(7):BR147-52.
